# Manual wheelchair downhill stability: an analysis of factors affecting tip probability

**DOI:** 10.1186/s12984-018-0450-3

**Published:** 2018-11-06

**Authors:** Louise Thomas, Jaimie Borisoff, Carolyn J. Sparrey

**Affiliations:** 1 School of Mechatronic Systems Engineering, Simon Fraser University, SFU Surrey Campus, 250-13450 102 Ave, Surrey, BC Canada; 2 British Columbia Institute of Technology, BCIT Centre for Applied Research & Innovation, 4355 Mathissi Pl, Burnaby, BC Canada; 3 International Collaboration on Repair Discoveries (ICORD), Blusson Spinal Cord Centre, 818 West 10th Avenue, Vancouver, BC V5Z 1M9 Canada

**Keywords:** Wheelchair stability, Mobility devices, Rigid body dynamics, Simulation, Motion capture

## Abstract

**Background:**

For people who use manual wheelchairs, tips and falls can result in serious injuries including bone fractures, concussions, and traumatic brain injury. We aimed to characterize how wheelchair configuration changes (including on-the-fly adjustments), user variables, and usage conditions affected dynamic tip probability while rolling down a slope and contacting a small block.

**Methods:**

Rigid body dynamic models of a manual wheelchair and test dummy were created using multi-body software (Madymo, TASS International, Livonia, MI), and validated with 189 experiments. Dynamic stability was assessed for a range of seat angles (0 to 20° below horizontal), backrest angles (0 to 20°), rear axle positions (0 to 20 cm from base of backrest), ground slopes (0 to 15°), bump heights (0 to 4 cm), wheelchair speeds (0 to 20 km/hr), user masses (50 to 115 kg), and user positions (0 to 10 cm from base of backrest). The tip classifications (forward tip, backward tip, rolled over bump, or stopped by bump) were investigated using a nominal logistic regression analysis.

**Results:**

Faster wheelchair speeds significantly increased the probability of tipping either forward or backward rather than stopping, but also increased the probability of rolling over the bump (*p* < 0.001). When the rear axle was positioned forward, this increased the risk of a backward tip compared to all other outcomes (*p* < 0.001), but also reduced the probability of being stopped by the bump (*p* < 0.001 compared to forward tip, *p* < 0.02 compared to rolling over). Reclining the backrest reduced the probability of a forward tip compared to all other outcomes (*p* < 0.001), and lowering the seat increased the probability of either rolling over the bump or tipping backwards rather than tipping forward (*p* < 0.001). In general, the wheelchair rolled over bumps < 1.5 cm, and forwards tipping was avoided by reducing the speed to 1 km/hr.

**Conclusions:**

The probability of forward tipping, corresponding to the greatest risk of injury, was significantly reduced for decreased speeds, smaller bumps, a reclined backrest, and a lower rear seat height. For wheelchairs with dynamic seating adjustability, when travelling downhill, on-the-fly adjustments to the seat or backrest can increase the likelihood of safely rolling over a bump.

## Background

It is estimated that approximately 1% of the population in developed countries require the use of a wheelchair [1, 2]. Each year, 3.3% of people who use wheelchairs in the United States are involved in serious accidents [3], sometimes resulting in traumatic brain injury, bone fractures, and concussions [4]. For active manual wheelchair users, the risk is even higher. Over a three year period from January 2006 to December 2008, 60.7% of people using manual wheelchairs (*n* = 56) reported tipping and falling at least once [5]. In the developed world, that equates to over 1.5 million manual wheelchair tips and falls every year.

The risk of a wheelchair tipping is related to its stability. Manual wheelchair static stability is defined by ISO 7176-1: 2014 as the angle at which a wheelchair and user tip over at rest [6]. However, there are currently no standards for determining manual wheelchair dynamic stability, that is, the risk of tipping while moving. Previous studies have considered manual wheelchair dynamic stability as the maximum speed that causes the wheelchair to stop rather than tip when rolling down a slope with a 5 cm bump at the end (while varying seat position and caster diameter) [7, 8]. Yet this fails to consider a range of obstacles that wheelchair users encounter, some of which they would be able to safely roll over. The lack of more comprehensive dynamic stability studies is likely due to the difficulties of experimentally controlling variables such as wheeling speed in a safe environment, and the considerable number of variables that affect the stability of a wheelchair in use. Such difficulties can be minimized by integrating computer simulations, validated with controlled experiments.

Rigid body dynamics are commonly used for biomechanical analyses of injuries [9] and falls [10], and are characterized by equations relating the kinematics of a system to the corresponding kinetic forces [11]. A key simplifying assumption, as suggested by the name, is the absence of deformation. This reduces the degrees of freedom, enabling problems to be solved without needing to calculate the stresses and strains in each segment. Compared to finite element analysis, rigid body dynamic simulations are therefore much more efficient and computationally inexpensive for analyzing large motions of bodies, making it an ideal method of studying wheelchair dynamics [12].

Our aim was to determine how fixed and spontaneous changes to a manual wheelchair configuration can affect the dynamic stability of the wheelchair rolling down a slope with a small bump at the end; a wheelchair skill that poses well-known safety concerns [13, 14]. Currently most manual wheelchairs are designed with a fixed frame [15], but more recent innovative designs allow users to adjust the seat angle and backrest angle ‘on-the-fly’ to suit their purposes [16]. These changes affect static stability by changing the centre of gravity of the system [17]. However, these changes are also likely to affect the inertia of the system and the resulting dynamic stability. The purpose of this study was to determine the effects of on-the-fly wheelchair configuration adjustments (seat angle and backrest angle), fixed wheelchair configuration changes (rear axle position), user variables (user mass, user positioning), and usage conditions (wheelchair velocity, slope of the ground, and bump height), on the dynamic tip probability of a wheelchair when moving down a slope.

## Methods

**Fig. 1:**
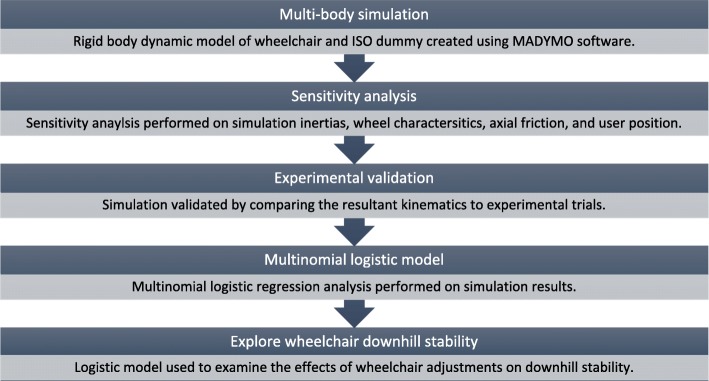
Methodology road map for estimating wheelchair dynamic tip probability

This study was comprised of a combination of simulations, experiments, and statistical analyses. First a rigid body simulation of the adjustable wheelchair was created and a sensitivity analysis was performed on that simulation. Simulations were then constructed and validated using matched experiments. Multinomial logistic parameter estimations were determined from the simulation results. Finally, the multinomial logistic model was explored to determine the effects of on-the-fly adjustability on downhill manual wheelchair stability (Fig. 1).

### Simulation

**Fig. 2:**
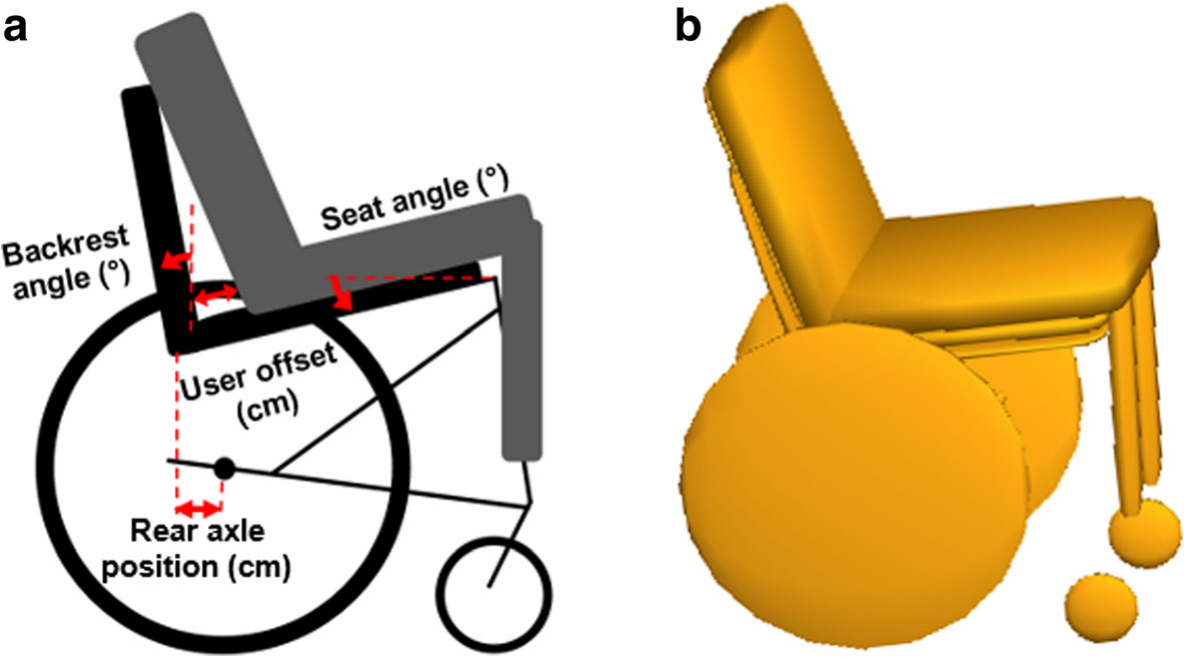
Diagram of wheelchair model. Variations were made to the wheelchair seat angle, backrest angle, rear axle position and user position (**a**), as well as user mass, wheelchair speed, ground slope, and bump height in the simulations. The Madymo model is shown on the right (**b**) Fig. 3 Experimental setup for testing wheelchair downhill stability

To quantify the effects of on-the-fly wheelchair configuration adjustments, fixed wheelchair configuration changes, user variables (Fig. 2a), and usage conditions on downhill stability, a rigid body dynamic model of a wheelchair (Fig. 2b, 16× 16, first generation Elevation™ model with 24 rear wheels and 5 casters, PDG Mobility, Vancouver, BC) and ISO standard test dummy were developed using MADYMO software (TASS International, Livonia, MI) and placed on a sloped ramp with a small obstacle at the end. The model was used to simulate a manual wheelchair and user rolling down a slope, over a small bump. The initial velocity of the wheelchair was assigned to the chair center of mass when the wheelchair front axles were 10 cm from the bump. The chair was then released to freely roll down the incline and impact the bump.

The wheelchair model was defined by seven components: the seat, backrest, front wheels (× 2), rear wheels (× 2), and frame. The point mass and inertia of each of these components were taken from a CAD model provided by PDG Mobility (Table 1). The mass distribution of the CAD model had been previously validated by comparing the tipping angle to that of the physical wheelchair for both forward and backward static stability [17]. The initial dummy measurements were taken from a CAD model of a 250 lb. test dummy, the same one used for the experimental validation. When varying the user mass, segment masses and centre of gravities (CoGs) were calculated from ISO 7176-11 [18]. The dummy was rigidly attached to the chair in the simulations to prevent relative motion between the dummy and the chair.

**Table 1:** Mass and inertia for all wheelchair and dummy components included in model

	Mass (kg)	Inertia: I_xx_, I_yy_, I_zz_, I_xy_, I_yz_, I_xz_ (kg.m^2^)
Component		
Front wheels (×2)	0.38	0.0005, 0.0009, 0.0005, 0, 0, 0
Rear wheels (×2)	1.80	0.0670, 0.1323, 0.0670, 0.0023, 0, 0
Seat (inc. gas springs)	3.21	0.0645, 0.0529, 0.0892, 0, 0, − 0.0044
Backrest	1.24	0.0435, 0.0253, 0.0242, 0 0–0.0016
Wheelchair frame	3.19	0.1328, 0.1187, 0.2024, 0 0–0.0117
Total wheelchair mass	12.00	
Torso	62.80	0.9439, 0.6674, 1.3138, 0, 0, 0.0730
Thigh	42.16	0.9682, 0.5219, 1.2659, 0, 0, 0.0702
Legs (×2)	4.16	0.0182, 0.1022, 0.0871, 0, 0, 0.0163
Total dummy mass	113.28	

The loading characteristics of the rear wheels and casters, which define the compression response during contact, were calculated by measuring the static deflection of each wheel under masses ranging from 0 to 40 kg, and fitting a curve to the results. The unloading curve was defined as a percentage of the loading curve. For the rear wheels, this was calculated by measuring the reduction in bounce height of the wheels when they were dropped from heights of 15–30 cm, which was recorded and analyzed using motion capture. The mean unloading/loading ratio for the rear wheels was 0.810 (σ = 0.027). For the casters, the assembly was measured as a whole since the housing also has a significant effect on contact characteristics [19]. For the cases where the wheelchair was stopped by the bump during the experimental testing, the unloading percentage was calculated using the average distance the wheelchair rolled back up the slope after impact with the bump. Using this method, the mean unloading/loading ratio for the caster housing was 0.294 (σ = 0.145). The axial friction in the wheels were found experimentally by rotating each of the wheels and recording the deceleration using motion capture. The process was repeated three times for each wheel, with the frictional torque calculated from the wheels’ inertias and the resulting angular decelerations. The front wheels had a mean frictional torque of 0.000918 N/m, and the rear wheels 0.00263 N/m.

A sensitivity analysis was performed to determine the accuracy and sensitivity of various model inputs, including the inertia of each segment, wheel loading and unloading characteristics, axial frictions, and offsets between the user and the wheelchair backrest. Each parameter was altered independently at least 5 times for a set of simulations (66 trials), and evaluated by the number of simulation outcomes matching the experimental results. Additional simulations were run with variations to caster diameter (4″, 5″ and 6″). These were separate from the rest of the sensitivity analysis as the wheelchair caster diameter was known, but changes to that diameter (if different casters were used) would likely have a significant impact on the probability of rolling over. For these simulations, all other wheelchair configuration variables were held constant (seat angle 10°, backrest angle 10°, rear axle position 10 cm from the base of the backrest, a slope of 4.8°, user mass of 75 kg, and no offset between the user position and backrest), and the bump height was increased in increments of 1 mm until the wheelchair no longer rolled over the bump. This procedure was followed for 3 different speeds (1, 3, and 5 km/h).

### Experimental validation

**Fig. 3:**
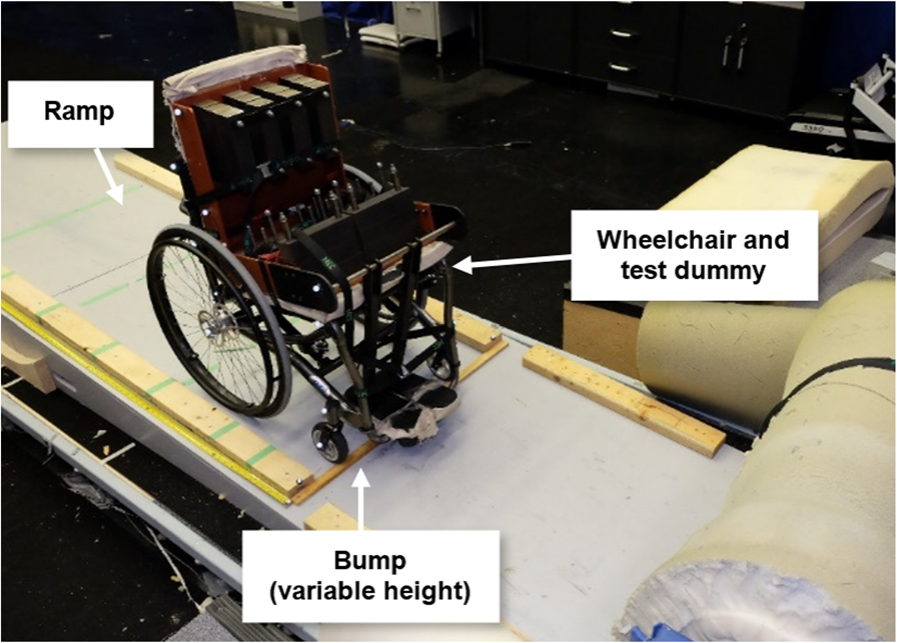
Experimental setup for testing wheelchair downhill stability

The model was validated by comparing simulations of the user and wheelchair rolling down a slope and into a bump to the kinematics of the physical wheelchair and test dummy, which was captured using 3D motion capture (Vicon, Oxford, UK). The dummy was strapped to the chair during testing to minimize relative motion between the dummy and the wheelchair, and padding was placed at the end of the ramp to minimise damage when forward tipping (Fig. 3).

The wheelchair was tested for a full-factorial combination of nine seat and backrest configurations (Table 2), two ramp angles (4.8° and 7.8°), three bump heights (1.3 cm, 1.9 cm, and 3.2 cm), and at least four speeds (up to 5.3 km/hr). This resulted in a total of 189 trials. The lower ramp angle was a 1:12 slope as recommended by the Americans with Disabilities Act [20], and the bumps were created from standard timber to represent small obstacles typical of activities of daily living. Speed was varied by changing the release distance from the bump to the front wheels. Wheelchair kinematic behaviour was classified into four categories; rolled over bump, stopped by bump, tipped forwards, or tipped backwards. These classifications were used to compare the simulations to the physical experimental results.

**Table 2:** Wheelchair seat and backrest configurations used for validation tests

Configuration type	Seat angle	Backrest angle
1	16.1° below horizontal	−1.0°
2		17.4°
3		34.7°
4	1.3° below horizontal	−1.0°
5		14.6°
6		29.0°
7	13.6° above horizontal	−1.0°
8		6.1°
9		17.6°

Seat angles ranged from 16.1° below horizontal to 13.6° above horizontal, and back angles ranged from vertical to a recline of 34.7°

### Analysis

Due to the number of variables, a Latin Hypercube experimental design [21, 22] was used to run 2000 variations of the validated model. The independent variables were the seat angle (0 to 20° below horizontal), backrest angle (0 to 20° from vertical), rear axle position (0 to 20 cm from base of backrest), slope of the ground (0 to 15°), bump height (0 to 4 cm), and speed of the wheelchair (0 to 20 km/hr), user mass (50 to 115 kg), and user position from base of backrest (0 to 10 cm). The geometry of the dummy model was constant for all user masses, and the CoG of the torso, thigh, and leg sections changed according to the wheelchair dummy standards [18]. The inertia values were scaled by the change in mass of each segment, and transformed using parallel axis theorem for changes in CoG locations. The observed dependent variable was the tip condition of the chair after impact with the bump. The final position of the wheelchair after impact with the bump was characterized as tipped forward, tipped backward, rolled over or stopped. A nominal logistic regression analysis was performed on the tip classifications using JMP software to determine the effects of the independent wheelchair configuration and user variables on the resulting tip behaviour (v13, SAS Institute, NC, USA). *P*-values less than 0.05 were considered significant, with results grouped by *p* < 0.001, *p* < 0.02 and *p* < 0.05.

## Results

### Simulation sensitivity analyses

The wheel unloading curve for the front casters had the greatest impact on model accuracy (Table 3). Rear wheel friction had an increased effect because, for the sensitivity analysis, speed was controlled by releasing the wheelchair from varied distances up the slope (the same as the experiment) and so axial friction affected impact speed. However, for the final simulations, an initial velocity was assigned to the wheelchair directly before hitting the bump, thus mitigating the effect of axial friction. User positioning also had a considerable effect on model sensitivity, highlighting the need to consider posture and user movement when configuring manual wheelchairs. For each inch increase in caster diameter, the maximum bump height that the wheelchair could successfully roll over increased by 2–3 mm (Table 4). For situations where the wheelchair could not roll over the bump, results differed depending on speed: for higher bumps, the wheelchair stopped when travelling at slower speeds (≤ 3 km/h), tipped forward when travelling at higher speeds (≥ 5 km/h). The effect of caster diameter on dynamic stability had been previously studied [7], and was not included in the main model as it is well known that larger diameter casters assist in rolling over higher bumps.

**Table 3:** Sensitivity of wheelchair model to set parameter changes

	Parameter variation	Percentage change in correct simulations
Torso inertia	50–150% of original	4.5%
Thigh inertia	50–150% of original	7.6%
Wheel unloading characteristics	50–150% of original	21.2%
Wheel loading characteristics	50–150% of original	6.1%
Rear wheel friction	50–150% of original	10.6%
Caster wheel friction	50–150% of original	1.5%
Position offset between user and base of backrest	±1.5 cm from original	6.1%
Position offset between user and top of backrest	±1.5 cm from original	10.6%

**Table 4:** Maximum bump height that the wheelchair rolled over for different caster diameters and speeds

Speed	Caster diameter		
	4 in	5 in	6 in
1 km/h	1.2 cm	1.5 cm	1.7 cm
3 km/h	1.7 cm	1.9 cm	2.2 cm
5 km/h	2.1 cm	2.4 cm	2.7 cm

### Validation with experiments

Of the 189 validation simulations performed, 168 (89%) achieved the same tip classification as the experimental results (Tables 5, 6 and 7). The most common occurrence was rolling over the bump (84 out of 189 experimental trials), while tipping backwards was least likely to occur (Table 5). Backwards tipping was also the least accurately modelled case, with a positive predictive value (PPV) of 64.3% (Table 6). The simulations were most accurate for low bumps (1.27 cm) and least accurate when the bump height was 1.91 cm (Table 7). The majority of trials rolled over the low bump, and were stopped or tipped forward for the high (3.18 cm) bump. The tip outcomes were more variable for the mid-sized bump.

**Table 5:** Experimental vs. simulation confusion matrix

Experimental result	Simulation result				
	Forward tip	Backward tip	Rolled over	Stopped	Total
Forward tip	28	2	–	6	36
Backward tip	–	9	1	1	11
Rolled over	3	2	78	1	84
Stopped	–	1	4	53	58
Total	31	14	83	61	189

**Table 6:** Classification statistics for simulations compared to experimental results

	Tip category			
	Forward tip	Backward tip	Rolled over	Stopped
Prevalence	0.190	0.058	0.444	0.307
Sensitivity	0.778	0.818	0.929	0.914
Specificity	0.980	0.972	0.952	0.939
PPV	0.903	0.643	0.940	0.869
NPV	0.949	0.989	0.943	0.961
F_1_ score	0.836	0.720	0.934	0.891

Rolling over the bump was the most common scenario, followed by being stopped by the bump. The F_1_ score was greatest for rolling over the bump, and least accurate for backward tips

**Table 7:** Comparison of simulation and experimental results, grouped by slope and bump height

Slope angle	Bump height	Sims correct	Sims incorrect	Discrepancies		Percentage correct
				Simulations	Experiments	
7.8°	1.3 cm	24	1	Rolled	Backwards tip	96.0
7.8°	1.9 cm	22	5	3x forward tip 2x backward tip	Rolled Rolled	81.5
7.8°	3.2 cm	24	3	Stopped Stopped Backward tip	Backward tip Forward tip Forward tip	88.9
4.8°	1.3 cm	33	1	Stopped	Rolled	97.1
4.8°	1.9 cm	33	6	4x rolled 2x stopped	Stopped Forward tip	84.6
4.8°	3.2 cm	32	5	3x stopped Backward tip Backward tip	Forward tip Forward tip Stopped	86.5

For 189 trials, 88.9% of the simulations gave the same results as the experiment

**Fig. 4:**
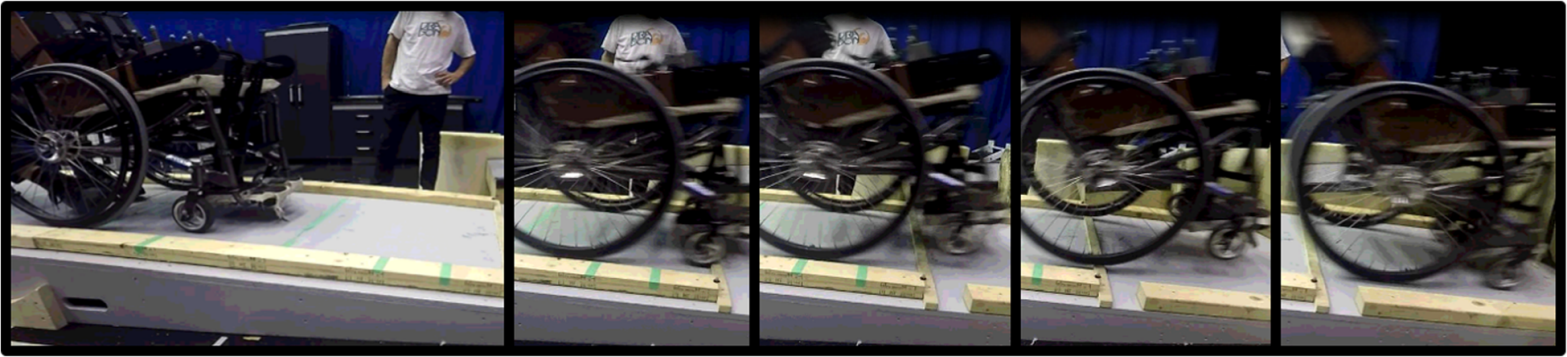
Experimental sequence of events for wheelchair rolling over a medium bump (1.91 cm) at 3.92 km/h.(1) wheelchair released on slope, (2) casters impact bump, (3) the momentum of the wheelchair causes the casters to launch over bump, (4) rear wheels impact bump while casters are still in the air, (5) wheelchair continues rolling down slope

**Fig. 5:**
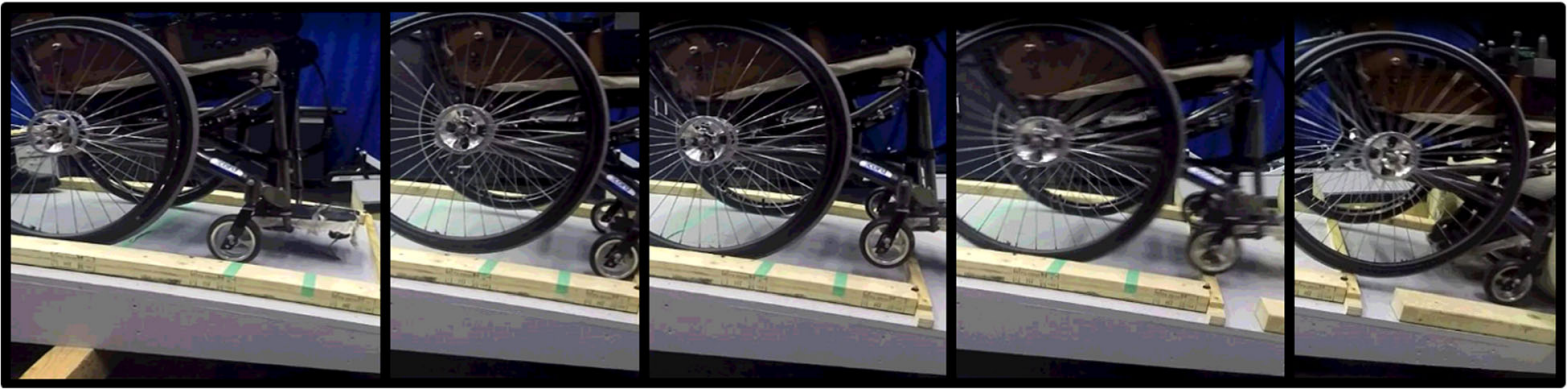
Experimental sequence of events for wheelchair rolling over a high bump (3.18 cm) at 2.59 km/h.(1) wheelchair released to roll down slope, (2) casters impact bump and rear wheels lift, (3) the rear wheels return to the ground, but the momentum causes the casters to lift, (4) casters clear bump, (5) the rear wheels follow, also clearing the bump

At higher speeds, the front of the wheelchair often became airborne on impact with the bump (Fig. 4). In some cases, this assisted in rolling over the bump, but also increased the probability of a backwards tip. Backwards tipping generally occurred when the wheelchair launched over the bump and the casters did not come down after clearing the bump. With the large test dummy, flex was observed in the wheelchair frame on impact with the bump, particularly to the backrest. For higher bumps, the wheelchair rolled over the bump using a rocking motion that popped the castors up (Fig. 5).

### Multinomial logistic model

The multinomial logistic parameter estimations (Table 8) showed bump height and speed were the most influential parameters on tip outcomes; rear axle position and backrest angle had the greatest effect of the wheelchair configuration variables. Speed had a significant effect on all tip classifications, and the backrest angle had a significant effect (*p* < 0.001) on all comparisons apart from ‘rolled vs stop’. Lowering the seat made the wheelchair significantly more likely to roll over the bump or tip backwards rather than tipping forwards.

**Table 8:** Multinomial logistic parameter estimations, with standard errors in brackets

	Forward tip vs Stop	Backward tip vs Stop	Rolled vs Stop	Backward vs Forward tip	Rolled vs Forward tip	Rolled vs Backward tip
Bump height (cm)	−0.127 (0.292)	−6.088*** (0.467)	−7.612*** (0.473)	−5.962*** (0.439)	−7.486*** (0.448)	−1.524*** (0.148)
Speed (km/hr)	2.311*** (0.235)	2.684*** (0.244)	2.851*** (0.244)	0.373*** (0.041)	0.540*** (0.040)	0.167*** (0.022)
Rear axle position (cm)	0.170*** (0.038)	0.547*** (0.049)	0.128** (0.042)	0.377*** (0.035)	−0.042 (0.024)	−0.419*** (0.032)
Backrest angle (°)	−0.119*** (0.035)	0.258*** (0.042)	0.042 (0.038)	0.377*** (0.031)	0.160*** (0.026)	−0.216*** (0.022)
Slope (°)	0.532*** (0.065)	0.439*** (0.068)	0.493*** (0.067)	−0.094** (0.034)	−0.039 (0.030)	0.054* (0.025)
User position (cm)	0.006 (0.073)	−0.375*** (0.084)	−0.170* (0.080)	− 0.382*** (0.054)	−0.176*** (0.047)	0.205*** (0.039)
Seat angle (°)	−0.059 (0.035)	0.086* (0.041)	0.029 (0.039)	0.145*** (0.026)	0.087*** (0.023)	−0.058** (0.019)
User mass (kg)	0.025** (0.011)	−0.010 (0.012)	0.005 (0.012)	−0.035*** (0.008)	−0.020** (0.007)	0.015** (0.006)

The first three columns use the ‘stop’ condition as the reference category, the next two use ‘forward tip’ as the reference category, and the final column uses ‘backward tip’ as the reference. In that way comparisons were made between all categories. Bump height and wheelchair speed were the most influential parameters, with the rear axle position and backrest angle having the greatest effect of the parameters directly relating to wheelchair configuration (*p < 0.05, **p < 0.02, ***p < 0.001)

The results of the logistic analysis, considering only linear terms, had a generalized R^2^ value of 0.908 and a misclassification rate of 10.2% (Table 9). The majority of simulations (1093 of 2000) rolled over the bump, and rolling over was accurately predicted by the logistic model 94.9% of the time (Table 10). Backwards tips were the most likely behaviour to be misclassified, and 37.9% of the simulations that tipped backwards were misclassified as rolling over. Being stopped by the bump was the least likely scenario, occurring for 7.55% of simulations with a model sensitivity of 92.1% (Table 10). With interaction terms included in the analysis, the generalized R^2^ value increased to 0.942 and the misclassification rate was reduced to 7.6%. Significant interaction effects with *p* < 0.001 were found for [speed]*[bump height], [rear axle position]*[bump height], [speed]*[rear axle position], [speed]*[slope], [backrest angle]*[rear axle position], [slope]*[bump height], and [user position]*[speed]. At the *p* < 0.02 level, interaction effects were also seen for [slope]*[backrest angle] and [user mass]*[user position].

**Table 9:** Confusion matrix for the logit model

Simulation result	Predicted logit model result				
	Forward tip	Backward tip	Rolled over	Stopped	Total
Forward tip	506	17	17	5	545
Backward tip	11	114	80	6	211
Rolled over	14	40	1037	2	1093
Stopped	8	1	3	139	151
Total	539	172	1137	152	2000

**Table 10:** Classification statistics for logit model compared to simulations

	Tip category			
	Forward tip	Backward tip	Rolled over	Stopped
Prevalence	0.270	0.106	0.547	0.076
Sensitivity	0.928	0.540	0.949	0.921
Specificity	0.977	0.954	0.869	0.993
PPV	0.939	0.663	0.912	0.914
NPV	0.973	0.933	0.913	0.994
F_1_ score	0.934	0.595	0.930	0.917

Overall there was a 10.2% misclassification rate when comparing the predicted result from the multinomial logistic analysis to the simulation results. Categories ‘forward tip’, ‘rolled over’, and ‘stopped’ all had F1 scores over 0.9, and ‘backward tip’ was the least accurate category with an F1 score of 0.595

**Fig. 6:**
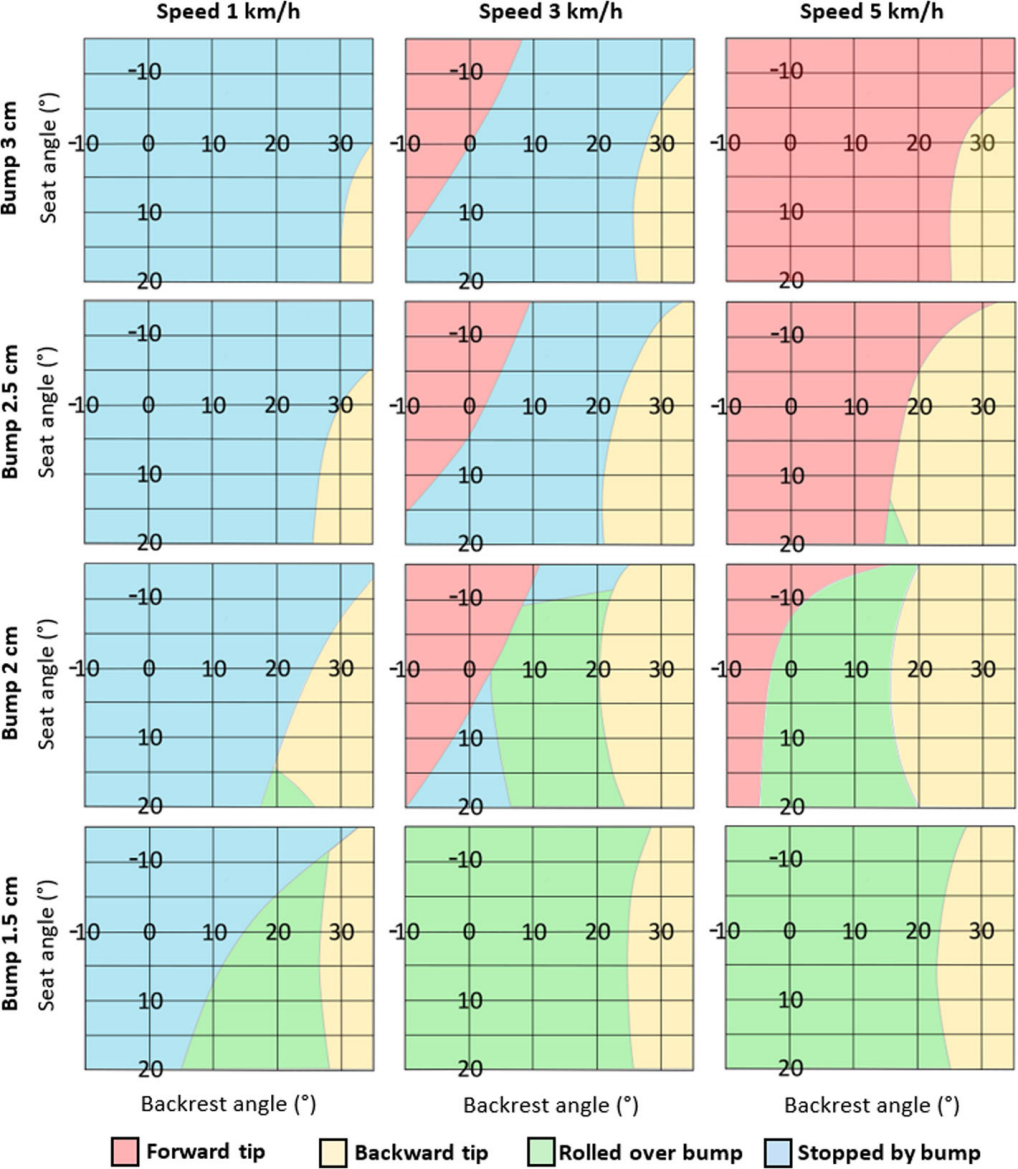
Expected wheelchair behaviour after rolling into/over a bump with respect to backrest and seat angles. Panels are grouped by speed and bump height

To explore the effects of on-the-fly adjustability on downhill stability, the expected wheelchair tip classifications from the logit model were plotted for different backrest angles, seat angles, speeds, and bump heights (Fig. 6). Rear axle position was held constant at 10 cm, slope was set to 4.8 degrees (equivalent to 1:12, a wheelchair standard for maximum ramp inclines), user mass set to 75 kg, and the user was positioned with no offset to the backrest. The plots show that bumps of 1.5 cm or less are unlikely to be an issue for manual wheelchairs to roll over, and forwards tipping over higher bumps can be avoided by reducing speed to 1 km/hr. Bumps of 2.5 cm and greater could generally not be rolled over regardless of variable configurations (except at higher speeds). For speeds of 1 km/h and 3 km/h, lowering the seat angle moved the expected outcomes of forward tipping or stopping to the safer results of stopping or rolling over. Similar results are shown for backrest recline, where a reclined backrest increases the likelihood of stopping rather than tipping forward and, for bumps < 2 cm, increases the probability of rolling over the bump instead of stopping. However, under greater backrest angle conditions, backwards tips are also possible.

## Discussion

Manual wheelchairs are an invaluable mobility aid for those that require them, but can pose a risk of tipping when traveling on sloped and uneven surfaces. Of manual wheelchair users that have experienced a fall, it is reported that 46.3% of falls were in the forward direction [21], which is also the tip direction most likely to result in a serious injury [22]. The top three self-reported causes of wheelchair related accidents are inexperience, uneven surfaces, and obstacles [5]. This study explored the stability of a manual wheelchair when wheeling down a slope and into a small bump using a combination of experiments and simulations. A comprehensive map of the effects of on-the-fly manual wheelchair configuration adjustments (seat angle and backrest angle), fixed wheelchair configuration changes (rear axle position), user variables (user mass, user positioning), and usage conditions (wheelchair velocity, slope of the ground, and bump height) on tip risk when wheeling downhill was determined. Bump height, wheeling speed and rear axle position were the most significant determinants of tipping probability, while on-the-fly adjustments to the seat angle and backrest angle could also change the outcome.

While standards exist for static stability [6], there are currently no standards for manual wheelchair dynamic stability. Previous studies considered dynamic stability rolling down a slope with a large (5 cm) bump at the bottom [7, 8, 23], where the outcome was either a stop or forwards tip. One such study showed that by moving the horizontal position of the seat (and therefore CoG) forward, the speed required to cause a forward tip decreases [8]. This agrees with our results, which show that forward movement of the CoG (by reducing the backrest angle or increasing user position offset from the backrest) increases the risk of a forward tip (Table 8).

A forward tip is the worst case scenario, and most likely to result in injuries requiring medical attention [22]. The parameters that had the greatest effect on forward tip probability were bump height, speed, and rear axle position. As the bump height increased, the speed required to roll over (assuming no torso movement) also increased. However, increasing speed also increased the risk of tipping rather than stopping. For lower bumps (≤2 cm), speed could be used to assist in overcoming obstacles, but this increases the risk of causing greater injury if a tip does occur. These results agree with prior work and highlight the importance of training wheelchair users to effectively navigate obstacles during downhill wheeling, including by adjusting their wheeling speed for different obstacles [13]. Lowering the seat significantly increased the probability of rolling over the bump and reduced the risk of a forward tip. When considering functional mobility, reclining the seat is also commonly used to improve balance and reach [24]. It is therefore recommended to lower the seat as far as possible, if the wheelchair includes this function, for downhill wheeling.

When wheeling downhill, the ideal outcome is for the wheelchair to roll over the bump. This occurred for 95% of simulations with a bump lower than 1 cm and backrest angle less than 20 degrees. However, if rolling over is not possible, it is much better for the wheelchair to be stopped by the bump rather than tip. In general, encountering a bump at 1 km/h (slow speed) allowed the user to safely stop without tipping. On level ground, comfortable propulsion speeds range from 3.7 km/h [25] to 4.6 km/h [26], with downhill wheeling sometimes faster. For terrain with bumps these speeds may become unsafe, thus for controlled wheeling the user may be required to slow down. Common obstacles encountered when wheeling downhill include potholes, rocks, and differences in pavement height, most of which are unlikely to be more than 2 cm in height. Wheelchair users can overcome higher obstacles such as curbs using torso rotation and controlled wheelies [27]. A similar type of movement was shown in Fig. 5, where the wheelchair pitched back and forth over the high bump. User movements (such as balancing in a wheelie) could be used in addition to configuration changes and speed to further improve downhill stability over bumps.

Reclining the backrest increased the probability of rolling over the bump or stopping rather than tipping forward. This did increase the risk of a backwards tip, but this was the least common outcome (5.8% of experiments and 10.6% of final simulations), was only an issue at very high backrest angles typically not used during active wheeling, and has been shown to be less dangerous than a tip forward [22]. The angle of the backrest can be the difference between a forward tip, being stopped by the bump, rolling over, or tipping backward (Fig. 6). A reclined backrest assists in maneuvering over bumps, but once the angle is more than 20 degrees there becomes a risk of tipping backward. This is similar to the static stability of the wheelchair, where a more reclined backrest enables the wheelchair to be more maneuverable, but less stable [17]. For wheelchairs without adjustable backrests, the user will usually have to perform a wheelie to go down steep inclines [13], which many users find unsafe or are unable to perform [28]; reclining the backrest may negate the need to do this. However, users with fixed framed wheelchairs may also benefit from knowing the quantified effects of backrest and seat angle on dynamic downhill stability, as it could assist in selecting the correct configuration for daily usage conditions. Depending on individual stability requirements, adjusted results from this study could be used to create guidelines to inform users and therapists of customized stability limits and maneuverability changes resulting from different wheelchair configurations.

User positioning has been previously shown to have a significant effect on stability [29]. When the user’s pelvis was positioned at an offset from the backrest, the probability of tipping backward was significantly reduced in comparison to all other behaviours. However, the probability of tipping forward rather than rolling over was also increased. For users that sit with their hips forward from the base of the seat, configuring the wheelchair with the rear axle further forward can permanently reverse the ensuing stability effects, or a reclined backrest could be used to temporarily adjust the stability as needed. As suggested by the Wheelchair Skills Training Program Manual, users should therefore be encouraged to reposition themselves as far back in the wheelchair as possible during downhill wheeling [13] to reduce the risk of a forward tip.

In general, configuration changes that made the wheelchair more likely to roll over the bump (lowering the seat, reclining the backrest, moving the rear axle forward) did so by shifting the system CoG towards the rear axles. On level ground, backward shifts in the CoG position also increase maneuverability [30]. The position of the rear axle had the greatest effect on tip response at slower speeds and when the bump was between 1.5 and 2.5 cm. For these cases, the outcome was less predictable and the position of the rear axle could be the deciding factor of whether the wheelchair tipped or rolled over. Moving the rear axle further forward made the chair more likely to tip backwards; interestingly, it also slightly increased the probability of rolling over the bump or tipping forwards rather than being stopped. Rolling over probability was likely increased due to shifting the CoG towards the rear axle, which reduced the load on the front wheels, making it easier for them to clear the bump. The increase in forward tipping probability may be owing to the weight of the rear wheels shifting the CoG forwards in relation to the front wheels. The effect of wheel position on dynamic rolling stability highlights the need for therapists and industry professionals to properly configure the wheelchair for each particular user. These results relate to previous research on manual wheelchair static stability, which showed that forward movements of the rear axle reduced stability, but increased maneuverability for a straight trajectory (defined as minimizing rolling resistance) [17]. It also suggests an opportunity for future designs offering a rear axle (or CoG) ‘on-the-fly’ adjustment capability that could significantly improve wheeling stability on slopes.

Changes in wheelchair configuration that affect downhill stability will also affect maneuverability and biomechanical demand during manual wheelchair propulsion [24, 31, 32]. The mobility of a manual wheelchair is a function of both the biomechanics of the user and the dynamics of the wheelchair itself. For situations where the user is pushing the chair (i.e. most dynamic cases apart from wheeling downhill), reducing rolling resistance and improving push biomechanics are important for minimizing the risk of upper limb overuse injuries [31, 33–35]. Increasing the load on the rear wheels reduces rolling resistance for straight trajectories [32], such as the modelled case of wheeling downhill, but does so at the cost of reducing rear stability [17, 30]. In addition to mechanical advantages due to reduced rolling resistance, shifting the rear axle forward increases the biomechanical push angle and shoulder ROM [24], and decreases needed muscle activity for the triceps, anterior deltoids and biceps [36]. The optimal seat angle for propulsion efficiency is still unknown [24], but a horizontal seat has been linked to the development of shoulder pain [37]. However, small changes in system tilt and seat to backrest angle (up to 10°) did not show any effect on joint angles or shoulder moments in manual wheeling [38]. Though a lower seat may be biomechanically superior for wheeling, an elevated seat can improve daily tasks such as transferring and reaching, and provide psychosocial benefits such as reducing eye to eye level discrepancies with others [39]. In daily life, wheelchair users perform a variety of maneuvers including movements forward, backward, turning, and accelerating. During straight motion the majority of propulsion energy is converted to translational energy, with some rotational kinetic energy for the wheels and casters, but during turning up to 71% of the system energy is converted to turning kinetic energy [38]. Therefore it is also important to consider multi-directional wheelchair maneuverability when evaluating complete wheelchair performance, where an increase in rear wheel loading corresponds to an increase in resistive forces due to turning [40]. Better dynamic wheelchair performance is likely a balance between stability, rolling resistance, and turning resistance, with the optimal configuration dependent on task specific requirements. Thus, the ability to change wheelchair configurations on-the-fly to emphasize different performance advantages may be beneficial to wheelchair users.

Our analysis demonstrated that on-the-fly adjustments to wheelchair configurations can improve downhill wheeling stability; however, the dominant factors in determining tip risk were bump height, wheeling speed and rear axle position which are not affected by on-the-fly alterations. Furthermore, an incorrectly positioned adjustable wheelchair can decrease stability. Therefore, training users to effectively use on-the-fly adjustments and defining the limits of operation will be important for optimizing the potential stability benefits of the technology. The results of the analysis also show that backrest angle had a greater effect on downhill rolling stability than seat angle. As a result, a chair with an adjustable backrest alone [42] could provide most of the potential downhill wheeling stability benefits observed in this study.

### Strengths and limitations

Computational models are an efficient method for studying wheelchair dynamics, however they are limited by model input accuracy [12]. The use of passive dummy models is a particular limitation, as it disregards any active movements of the user. For the case of rolling down a slope this is not a major issue as users are advised to maintain their weight towards the rear of the wheelchair when descending [13]. However, when navigating obstacles and for other situations where the user actively changes their position, future models will need to be modified to simulate user activity. Since the mass of the user represents the majority of the system mass, dummy stature is another limitation. The ISO dummies used represent the average stature of a wheelchair user [18], but individual variations may affect model accuracy by changing the mass distribution and therefore the inertial characteristics and centre of mass of the user.

Discrepancies between the simulation and experimental results were likely due to the model being highly sensitive to the material properties of the wheels, and limitations in the method of measuring axial friction of the wheels. This is demonstrated by the increased sensitivity of the model to the wheel unloading characteristics (Table 3). Rigid body models are unable to fully capture the dynamics of collisions [41]. Since some deformation occurs on impact with the bump, finite-element methods could improve the accuracy of the tire contact calculations. Including tire deformation would also allow the rolling resistance of the wheelchair to be more accurately modelled. However, using finite element analysis in the model would greatly increase computational time and limit the number of simulations that could feasibly be run. The measured physical properties of the wheelchair were another possible source of error in the model. In particular, the accuracy of the wheel contact characteristics and the axial friction were limited by the methods used to measure them. Since the loading of the wheels were measured statically, they would not precisely match the dynamic loading characteristics during a collision. Measuring the dynamic loading of the wheels was outside the scope of this study. Using an unloaded axial friction load was also a limitation, but provided a reasonable approximation. Estimating friction coefficients from the deceleration of the wheels resulted in a less accurate model than using the friction loads from the unloaded wheel.

## Conclusion

A combination of skills training and dynamic wheelchair adjustability could greatly improve user safety when wheeling over obstacles. The most significant factors for downhill wheeling stability were bump height, speed, and rear axle position. Increased speed can be used to overcome smaller bumps where the user is confident they will not tip, but for larger bumps a more controlled method should be employed. Generally, this will involve skilled user movements such as balancing in a wheelie. However, the need for this maneuver could be negated by changing the CoG of the wheelchair system using on-the-fly wheelchair seat and backrest adjustability. The significant impact of rear axle position on both stability and wheelchair maneuverability also suggests an opportunity for the future development of wheelchairs with dynamically adjustable rear axles. This would enable users to optimize the balance between wheelchair stability and maneuverability as required throughout the day. Having developed a validated computer simulation of wheelchair tip dynamics, future research could include the effects of user movements on manual wheelchair stability and maneuverability. Since the weight of user constitutes such a large proportion of the total system, small movements in user position may have a large effect on the dynamics as a whole. This can be comprehensively explored using the developed simulation methods. This also underlines the importance of effective wheelchair skills training [13] in combination with good wheelchair design for safe and reliable wheelchair use. Consequently, manual wheelchair dynamics need to be analyzed as a complex system with interactions between the wheelchair itself, the user, and the environmental conditions.

On-the-fly adjustments to the seat and backrest could be used in certain situations to reduce the probability of tipping and/or increase the probability of rolling over a bump. The quantified general downhill rolling stability results could also be used to guide the configuration of fixed-frame wheelchairs or those with adjustable backrests only [42] to define more optimal operating limits. For wheelchairs with dynamic seat and backrest adjustability, when travelling downhill the seat should be lowered as far as possible to increase the likelihood of safely rolling over a bump. Reclining the backrest may also help in overcoming obstacles, but should be adjusted with caution as reclining will also increase the probability of a backwards tip.
